# Prevalence and Correlates of Steatotic Liver Disease Among People With HIV in Uganda

**DOI:** 10.1093/ofid/ofag325

**Published:** 2026-05-27

**Authors:** Prossy Bibangambah, Moses Acan, Brian Ghoshhajra, Geoffrey Erem, Rita Nassanga, Andrew Ochieng, Vikas Thondapu, Michael T Lu, Mangun Randhawa, Angelo Takigami, Zahra Reynolds, Flavia Atwiine, Edna Tindimwebwa, Rebecca F Gilbert, Susanne S Hoepner, Eliza Passell, Shruti Sagar, Yao Tong, Ntobeko A B Ntusi, Judy Hahn, Thomas Gaziano, Christopher T Longenecker, Alexander C Tsai, Jennifer M Manne-Goehler, Stephen Asiimwe, Samson Okello, Mark J Siedner

**Affiliations:** Department of Radiology, Mbarara University of Science and Technology, Mbarara, Uganda; Department of Radiology, Mbarara University of Science and Technology, Mbarara, Uganda; Department of Radiology, Harvard Medical School, Boston, Massachusetts, USA; Department of Radiology, Massachusetts General Hospital, Boston, Massachusetts, USA; Department of Radiology, Makerere University, Kampala, Uganda; Department of Radiology, Nsambya Hospital, Kampala, Uganda; Department of Radiology, Makerere University, Kampala, Uganda; Department of Radiology, Nsambya Hospital, Kampala, Uganda; Department of Radiology, Nsambya Hospital, Kampala, Uganda; Department of Radiology, Mulago National Referral Hospital, Kampala, Uganda; Department of Radiology, Yale University School of Medicine, New Haven, Connecticut, USA; Department of Medicine, Harvard Medical School, Boston, Massachusetts, USA; Department of Medicine, Massachusetts General Hospital, Boston, Massachusetts, USA; Department of Medicine, Massachusetts General Hospital, Boston, Massachusetts, USA; Department of Medicine, Massachusetts General Hospital, Boston, Massachusetts, USA; Department of Medicine, Massachusetts General Hospital, Boston, Massachusetts, USA; South African Medical Research Council, South Africa and University of Cape Town, South Africa; Department of Medicine, University of California, San Francisco, California, USA; Department of Medicine, Massachusetts General Hospital, Boston, Massachusetts, USA; Department of Medicine, Harvard Medical School, Boston, Massachusetts, USA; Department of Medicine, Massachusetts General Hospital, Boston, Massachusetts, USA; Department of Medicine, Massachusetts General Hospital, Boston, Massachusetts, USA; Department of Medicine, Massachusetts General Hospital, Boston, Massachusetts, USA; Department of Medicine, Massachusetts General Hospital, Boston, Massachusetts, USA; South African Medical Research Council, South Africa and University of Cape Town, South Africa; Department of Medicine, University of California, San Francisco, California, USA; Department of Medicine, Harvard Medical School, Boston, Massachusetts, USA; Department of Medicine, Brigham and Women's Hospital, Boston, Massachusetts, USA; Department of Global Health, University of Washington, Seattle, Washington, USA; Mbarara University of Science and Technology, Mbarara, Uganda; Department of Psychiatry, Harvard Medical School, Boston, Massachusetts, USA; Department of Psychiatry, Massachusetts General Hospital, Boston, Massachusetts, USA; Harvard T.H. Chan School of Public Health, Boston, Massachusetts, USA; Department of Medicine, Brigham and Women's Hospital, Boston, Massachusetts, USA; Harvard T.H. Chan School of Public Health, Boston, Massachusetts, USA; Kabwohe Clinical Research Center, Kabwohe, Sheema, Uganda; Department of Community Health, Mbarara University of Science and Technology, Mbarara, Uganda; Harvard T.H. Chan School of Public Health, Boston, Massachusetts, USA; Department of Medicine, Mbarara University of Science and Technology, Mbarara, Uganda; Department of Epidemiology, University of North Carolina, Chapel Hill, North Carolina, USA; Department of Medicine, Harvard Medical School, Boston, Massachusetts, USA; Department of Medicine, Massachusetts General Hospital, Boston, Massachusetts, USA; Department of Medicine, Mbarara University of Science and Technology, Mbarara, Uganda; Africa Health Research Institute, KwaZulu-Natal, South Africa

**Keywords:** fatty liver disease, HIV, nonalcoholic fatty liver disease, steatotic liver disease

## Abstract

**Background:**

Liver disease is an important cause of morbidity and mortality among people with HIV (PWH). Steatotic liver disease (SLD) is a leading cause of liver disease among PWH in the Global North. However, there is a dearth of data on the epidemiology of SLD in sub-Saharan Africa.

**Methods:**

We analyzed computed tomography images from a completed cohort study composed of ambulatory people with and without HIV. We defined SLD as 1 or more of the following: mean liver attenuation (density) <40 Hounsfield units, mean liver to mean spleen attenuation ratio <1, or a Hounsfield unit difference <1 between the mean attenuation values of the liver and spleen. We fit multivariable regression models to evaluate the association of HIV with SLD. In a subanalysis to explore HIV-specific effects, we excluded those who reported high-risk alcohol consumption.

**Results:**

A total of 579 participants had interpretable liver images. The prevalence of SLD was low overall (22/579, 3.8%) and higher among PWH as compared with people without HIV (5.4% vs 2.3%, *P* = .056). In multivariable regression models, PWH undergoing antiretroviral therapy were more likely to have SLD (adjusted odd ratio, 2.79; 95% CI, 1.10–7.09; *P* = .031), even after exclusion of people with high-risk alcohol consumption (adjusted odd ratio, 2.96; 95% CI, 1.00–8.70; *P* = .048).

**Conclusions:**

We found a low overall prevalence of SLD in Uganda, but older PWH undergoing antiretroviral therapy had a higher prevalence of SLD than those without HIV. Variation in the prevalence of SLD in the African region may be due to the variability in the methods used.

## Teaser

Among older adults in rural Uganda, noncontrast computed tomography showed a low overall prevalence of steatotic liver disease, but people with HIV had higher odds than people without HIV, independent of alcohol misuse and metabolic risk factors after adjustment.

In the Global North, liver disease is an important cause of morbidity and mortality among people with HIV (PWH) and is responsible for approximately 1 in 6 HIV-related deaths [1]. One of the major manifestations of liver disease is steatotic liver disease (SLD) presenting as metabolic dysfunction–associated steatotic liver disease (MASLD), formerly known as nonalcoholic fatty liver disease. Other forms of SLD include alcohol-related/associated liver disease and metabolic- and alcohol-related/associated liver disease, an overlap of MASLD and associated liver disease [2].

There are multiple mechanisms by which PWH may be at risk for SLD. HIV infection has been directly associated with an increased risk for SLD, possibly due to persistent immune activation and resulting insulin resistance [3, 4]. Yet, PWH may be at increased risk of developing SLD due to drug toxicity, coinfections with viral hepatitis, and alcohol consumption [1, 5–8]. Obesity, diabetes mellitus, hyperglycemia, and hypertriglyceridemia, all prevalent among PWH, are also recognized components of MASLD [9, 10]. Moreover, integrase inhibitor–based antiretroviral therapy (ART) regimens, which now predominate as first-line HIV therapy globally, are associated with weight gain [11, 12] and are hypothesized to contribute to risk of SLD [13, 14].

In Africa, there is a dearth of data on the epidemiology of SLD [15]. With 9.1 million PWH in sub-Saharan Africa aged >50 years expected by the year 2040 [16], the intersecting noncommunicable disease epidemic [17], and the rapid transition to dolutegravir-based ART regimens, there is a pressing need to better understand the epidemiology of SLD in the region. To address this gap in knowledge, we sought to estimate the prevalence of SLD in a mixed cohort of PWH and a comparator group of age- and sex-similar people without HIV (PWoH) in the same clinic catchment areas in Uganda.

## METHODS

### Study Population and Inclusion Criteria

We conducted a cross-sectional analysis of data from the Epidemiology of Coronary Artery Disease Among People With HIV in Rural Sub-Saharan Africa (CAD) study. The CAD study comprised ambulatory PWH aged at least 40 years undergoing ART for a minimum of 3 years and an age- and sex-similar comparator group of PWoH. The study enrolled participants from 2 sites in southwestern Uganda between 2018 and 2023 [18]. At both sites, study participants were recruited first from HIV clinics (Mbarara Regional Referral Hospital HIV clinic and Kabwohe HIV Clinic); then, PWoH comparators who were age and sex similar (by quartile of the HIV-infected subgroup) were selected from population census data in villages within the clinic catchment area as previously described [19–21].

Participants were excluded if they had contraindications to contrast-enhanced computed tomography (CT) imaging that was conducted by the parent study. These criteria were (1) a confirmed estimated glomerular filtration rate <60 mL/min/1.73 m^2^ as calculated by the Chronic Kidney Disease Epidemiology Collaboration equation [22, 23] and/or (2) pregnancy confirmed by urine β-HCG testing among all women aged <60 years.

### Data Collection

Participants completed questionnaires about sociodemographic characteristics, medical history, and alcohol misuse using the Alcohol Use Disorders Test for Consumption (AUDIT-C) [24–26]. Trained research assistants measured body weight using standardized scales (762; seca), height using roll-up measuring stadiometers (206; seca), resting blood pressure using digital sphygmomanometers (Omron Healthcare Inc), and hip and waist circumference [27]. Study phlebotomists collected blood into EDTA tubes for point-of-care hemoglobin A_1c_ (DCA Vantage; Siemens), liver chemistries (*c*4000; Abbott Architect), serum lipids (1000*i*; Abbott Architect), hepatitis B testing (lateral flow assay; SD Bioline), full blood count (XN 350 model, 5-part hematology analyzer; Sysmex), CD4+ T-cell count (PIMA CD4 analyzer; Abbott), and HIV-1 RNA viral load (GeneXpert GX-IV-Module; Cepheid).

### CT Procedures

All eligible study participants underwent coronary CT angiography with a 128-slice single-source CT scanner (Siemens SOMATOM Definition AS; Siemens Healthineers) at Nsambya Hospital in Kampala. The coronary CT angiography protocol includes full field-of-view noncontrast CT images of the chest for evaluation of calcium scoring that includes part or all of the liver and spleen. Nsambya Hospital sent the acquired DICOM images to Massachusetts General Hospital for review of completeness and adherence to acquisition protocol. All study images were interpreted for clinical purposes by Ugandan certified radiologists, and referrals for follow-up care were made as appropriate.

### Liver and Spleen Attenuation Measurement

We used noncontrast axial CT images to measure fatty infiltration of the liver using standardized techniques [28]. In brief, we obtained Hounsfield unit (HU) attenuation values of the liver and spleen on noncontrast axial CT images using a circular region of interest (>2.0 cm^2^), avoiding vessels, ducts, and any present masses and calcifications. We collected data from 3 regions of interest on 3 slice images of the peripheral right hepatic lobe and spleen. The average of these 3 areas for the liver and spleen was calculated. In images in which the spleen was not interpretable, the attenuation values of only the liver were used. We defined SLD in participants meeting 1 or more standardized criteria [29–31]: a mean liver attenuation <40 HU, a mean liver to mean spleen attenuation ratio <1, or a HU difference <1 between the mean attenuation values of the liver and spleen. Two radiology readers (P. B. and V. T.) read liver and spleen attenuation measurements on 49 randomly selected participants to assess interreader reliability (κ = 0.83) prior to completion of all scans by a single reader (P. B.).

### Statistical Analysis

Our primary outcome of interest was SLD. Our primary exposure of interest was HIV serostatus. We first summarized participants’ clinical and demographic characteristics as frequency (percentage) for categorical variables and means (standard deviations) for continuous variables. We compared PWH vs PWoH on these characteristics, using the *t* test for continuous variables and χ^2^ or Fisher exact test for categorical variables, as appropriate. We next estimated the crude prevalence of SLD by HIV serostatus and then, in secondary analyses, by previously reported standardized indices of liver fibrosis, including the Fibrosis-4 (FIB-4) index (positive >3.25) [32] and aspartate aminotransferase to platelet ratio index (APRI; positive ≥1.5) [33]. In further analyses, we estimated the prevalence of probable MASLD (those with steatosis in the absence of alcohol misuse and hepatitis B infection) and metabolic- and alcohol-related/associated liver disease. We then fitted logistic regression models with SLD as the primary outcome of interest and HIV serostatus as the primary explanatory variable of interest, with and without the following potential confounders: age (scaled so that the estimated regression coefficients or odds ratios could be interpreted as the association per 10 years), sex, non–high-density lipoprotein (non-HDL) cholesterol (per 10 mg/dL), waist circumference (per centimeter), and hemoglobin A_1c_ (per 1%). Multivariable regression models included covariates that achieved significance as determined by *P* < .25 in univariable models. In sensitivity analyses, to increase specificity for probable MASLD as our outcome, we excluded 30 (10.8%) PWH and 53 (17.7%) PWoH who met criteria for alcohol misuse, as defined by an AUDIT-C score ≥4 among men and ≥3 among women [24–26]. We conducted all analyses using Stata version 15 (StataCorp).

### Ethical Considerations

The CAD study procedures were approved by the institutional review boards at the Mbarara University of Science and Technology (05/06-18), Mass General Brigham (2018P001037/MGH), and Nsambya Hospital. We received clearance to conduct the study from the Uganda National Council for Science and Technology (HS267ES). All participants provided written informed consent.

## RESULTS

**Figure 1.**
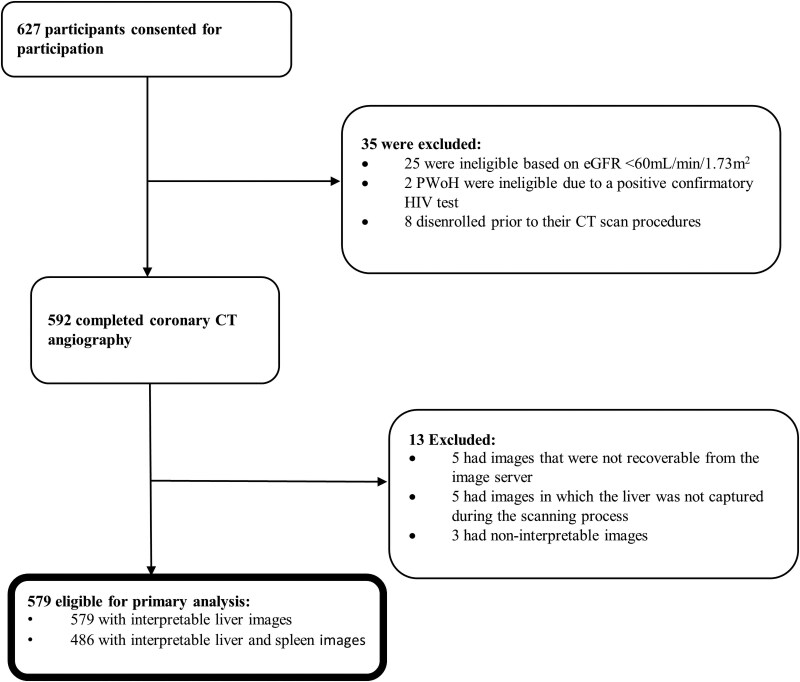
Study flow diagram. Abbreviations: CT, computed tomography; eGFR, estimated glomerular filtration rate; PWoH, people without HIV.

Of the 627 participants who consented for participation in the CAD study, 25 (4%) were ineligible based on estimated glomerular filtration rate <60 mL/min/1.73 m^2^; 2 (0.3%) PWoH were ineligible due to a positive confirmatory HIV test result; 8 (1.3%) disenrolled prior to their CT scan procedures; 5 (0.8%) had images that were not recoverable from the image server; 5 (0.8%) had images in which the liver was not captured during the scanning process; and 3 (0.5%) had noninterpretable images, leaving 579 participants with noncontrast CT scan images for evaluation in the SLD substudy analysis. Among these, 579 (100%) had interpretable liver images while 486 (84%) had interpretable spleen images for calculation of the liver-spleen ratio (Figure 1).

The cohort was nearly equally divided by HIV serostatus and sex with a mean age of 57.7 years (SD, 6.7; Table 1). PWH and PWoH were similar in terms of demographic and clinical characteristics, except for systolic blood pressure (126.5 vs 122.9 mm Hg, *P* = .028) and non-HDL cholesterol (116.8 vs 109.4 mg/dL, *P* = .018), which were moderately higher in PWoH. The proportion of people with alcohol misuse was also higher among the PWoH (17.7% vs 10.8%, *P* = .018).

**Table 1.** Cohort Characteristics

Characteristic	PWoH (n = 300)	PWH (n = 279)	*P* Value
Age, y^a^	58.0 (7.0)	57.4 (6.4)	.306
Female	147 (49.0)	135 (48.4)	.883
Waist circumference, cm^a^	85.0 (11.1)	85.6 (12.5)	.584
Body mass index, kg/m^2^			.034
<18.5	27 (9.0)	27 (9.7)	
18.5 to <25	146 (48.7)	163 (58.4)	
25 to <30	80 (26.7)	64 (22.9)	
>30	47 (15.7)	25 (9.0)	
Blood pressure, mm Hg^a^			
Systolic	126.5 (20.0)	122.9 (19.1)	.028
Diastolic	79.0 (11.5)	78.8 (12.4)	.758
Hemoglobin A_1c_, %^a^	5.8 (1.0)	5.8 (1.3)	.869
Non-HDL cholesterol, mg/dL^a^	116.8 (37.1)	109.4 (37.6)	.018
Hepatitis B surface antigen			.533
Negative	296 (98.7)	273 (97.8)	
Positive	4 (1.3)	6 (2.2)	
AUDIT-C			.018
Negative	247 (82.3)	249 (89.3)	
Positive	53 (17.7)	30 (10.8)	
FIB-4^b^			<.001
Mild/no fibrosis	125 (54.1)	47 (30.9)	
Indeterminate	96 (41.6)	92 (60.5)	
Significant fibrosis	10 (4.3)	13 (8.6)	
APRI^c^			.007
Mild/no fibrosis	214 (92.6)	126 (82.9)	
Indeterminate	16 (6.9)	24 (15.8)	
Significant fibrosis	1 (0.4)	2 (1.3)	
Liver attenuation, HU^a^	62.3 (9.1)	61.2 (9.8)	.178
Liver/spleen attenuation			.136
>1	239 (98.0)	231 (95.5)	
<1	5 (2.0)	11 (4.5)	
Liver-spleen attenuation difference			.158
>1	238 (97.5)	230 (95.0)	
<1	6 (2.5)	12 (5.0)	
Current CD4 count, cells/mm			
<350	…	50 (17.9)	
350–499	…	83 (29.8)	
≥500	…	146 (52.3)	
Viral load			
HIV-1 RNA <40 copies/mL	…	194 (71.1)	
Antiretroviral regimen			
Dolutegravir-containing regimen	…	232 (83.2)	

Data are presented as No. (%) unless noted otherwise.

Abbreviations: APRI, aspartate aminotransferase to platelet ratio index; AUDIT-C, Alcohol Use Disorders Test for Consumption; FIB-4, Fibrosis-4 index; HBV, hepatitis B virus; HDL, high-density lipoprotein; HU, Hounsfield unit; PWH, people with HIV; PWOH, people without HIV.

^a^Mean (SD).

^b^FIB-4: <1.45, no fibrosis; ≥1.45 to ≤3.25, indeterminate; >3.25, significant fibrosis.

^c^APRI: <0.5, no fibrosis; >0.5 to <1.5, indeterminate; ≥1.5, significant fibrosis.

**Figure 2.**
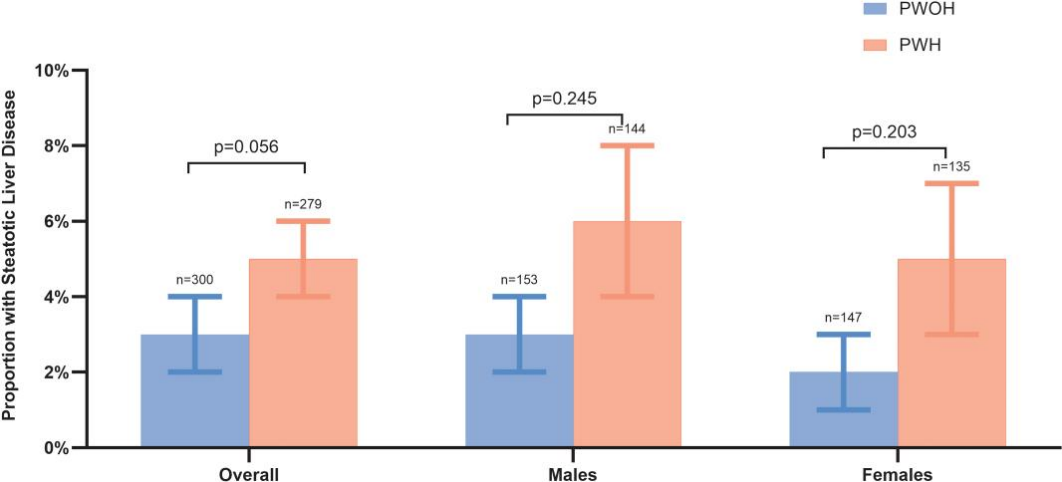
Crude prevalence of steatotic liver disease by HIV serostatus and sex. The bars display standard errors. PWH, people with HIV; PWOH, people without HIV.

PWH had a higher prevalence of liver fibrosis than PWoH as estimated by the FIB-4 index (8.6 vs 4.3, *P* < .001) and APRI score (1.3 vs 0.4, *P* = .007). The prevalence of hepatitis B virus in the overall cohort was 1.7% (10/579), with no difference by HIV serostatus. The crude prevalence of our primary outcome, SLD, was 3.8% overall (22/579) and marginally higher among PWH vs PWoH (5.4% [15/279] vs 2.3% [7/300], *P* = .056; Figure 2). The prevalence of SLD was higher among those with significant fibrosis as determined by a positive FIB-4 index (13%) and/or a positive APRI score (67%; Supplementary Figure 1), although the overall prevalence of significant fibrosis was low by these measures in this sample (Table 1). The prevalence of those with probable MASLD (those with steatosis in absence of alcohol misuse and hepatitis B infection) and metabolic- and alcohol-related/associated liver disease was 3.3% (16/487) and 3.7% (21/569) respectively.

In multivariable models, the following were associated with higher odds of SLD: HIV (adjusted odds ratio [AOR], 2.79; 95% CI, 1.10–7.09; *P* = .031), non-HDL cholesterol (AOR, 1.13; 95% CI, 1.02–1.25; *P* = .025), and increasing waist circumference (AOR, 1.04; 95% CI, 1.00–1.08; *P* = .039; Table 2). In secondary analyses restricted to those without evidence of alcohol misuse by AUDIT-C scores, the crude prevalence of SLD was 3.4% and remained modestly higher among PWH vs PWoH (4.8% vs 2.0%, *P* = .056). In multivariable models after removal of those with alcohol misuse, HIV (AOR, 2.96; 95% CI, 1.00–8.70; *P* = .048) and non-HDL cholesterol (AOR, 1.13; 95% CI, 1.01–1.27; *P* = .033) remained associated with the presence of SLD (Supplementary Table 1).

**Table 2.** Correlates of Steatotic Liver Disease

	Unadjusted Model		Adjusted Model^a^	
Factor	OR (95% CI)	*P* Value	OR (95% CI)	*P* Value
HIV serostatus				
PWoH	1 [Reference]			
PWH	2.38 (.96–5.92)	.063	2.79 (1.10–7.09)	.031
Age, each 10 y	0.76 (.38–1.51)	.435		
Female	0.87 (.37–2.05)	.756		
Non-HDL cholesterol, each 10 mg/dL	1.16 (1.05–1.28)	.003	1.13 (1.02–1.25)	.025
Waist circumference, each cm	1.05 (1.02–1.08)	.004	1.04 (1.00–1.08)	.039
Hemoglobin A_1c_, each %	1.08 (.81–1.45)	.598		
Blood pressure, each 10 mm Hg				
Systolic	1.06 (.86–1.30)	.598		
Diastolic	1.13 (.80–1.59)	.487		
Body mass index, kg/m^2^				
18.5 to <25	1 [Reference]			
<18.5	0.71 (.09–5.80)	.749		
25 to <30	2.51 (.95–6.64)	.064		
>30	2.21 (.64–7.56)	.205		
AUDIT-C				
Negative	1 [Reference]			
Positive	1.81 (.65–5.04)	.258		

Abbreviations: AUDIT-C, Alcohol Use Disorders Test for Consumption; HDL, high-density lipoprotein; OR, odds ratio; PWH, people with HIV; PWOH, people without HIV.

^a^Adjusted model: all variables that had a *P* value <.25 in univariate/unadjusted model.

## DISCUSSION

In a cohort of older adult PWH and PWoH in Uganda evaluated for SLD with CT imaging, we found a low crude prevalence of SLD (3.8%). However, notwithstanding the low overall prevalence, PWH undergoing ART had approximately 2 times the prevalence of disease, and in multivariable models adjusting for likely confounders, HIV remained associated with a doubling of the odds of having SLD. This finding persisted after exclusion of people with alcohol misuse, and it suggests that although SLD may be relatively rare in rural Uganda, HIV increases the risk, even after consideration of traditional risk factors.

Our results are similar to a growing body evidence suggesting that HIV may be associated with increased risk of SLD [34, 35]. Numerous mechanistic pathways have been implicated in the pathogenesis of SLD among PWH. First, although older ART medications are being phased out—such as stavudine and zidovudine, which cause metabolic changes including lipodystrophy—the current dolutegravir-based ART regimens have been associated with steatosis and weight gain, which increase the odds of progression to fibrosis [12, 36]. Second, PWH have an increased prevalence of risk factors for SLD, such as obesity and dyslipidemia, as well as comorbidities associated with SLD, including drug-induced liver injury and infections such as hepatitis B and C virus [4]. Third, the additional effects of HIV infection in altering the gut microbiome disrupts the “gut-liver axis,” which is believed to contribute to the development and progression of SLD [37–39]. Finally, persistent immune activation due to HIV infection has been associated with insulin resistance and SLD [3, 4]. Our data suggest that the elevated risk among PWH pertain to people in rural Uganda.

We observed a low prevalence of SLD in Uganda. When compared with other regional estimates, there is variation in the prevalence of SLD in the African region, which has ranged from 5% to 15% in the general population [40]. The limited data available suggest a higher prevalence of SLD among PWH as compared with those without HIV [34]. The variability in these estimates highlights the scarcity of population prevalence–based data from the region. Moreover, these variations may be due to the variability in the methods used to estimate SLD. One study in Uganda reported a prevalence of 12% when assessing SLD using a fatty liver index score ≥60 as a definition [41], whereas another study in South Africa estimated a prevalence of 21% through liver biopsy samples [34]. The fatty liver index has been extensively validated in the general population for evaluation of SLD [42–45]. While participants in our study were ambulatory with no known history of liver disease and with a mean age of 58 years, the study using the fatty liver index was composed of a relatively younger sample of participants, aged 35 to 49 years (mean, 41), from a mostly rural population-based cohort survey of 1463 participants. By contrast, the study in South Africa estimated SLD using biopsy samples taken from 262 participants with known liver disease of unknown etiology, which makes direct comparison with our population-based design challenging. Moreover, its use of liver biopsy and histology, which is more sensitive than imaging and remains the gold standard for diagnosis of SLD, may have further contributed to the higher prevalence reported in that study [46]. Additionally, studies have shown regional variation, with southern Africa having a higher prevalence of obesity and increased risk of weight gain for those prescribed dolutegravir in comparison with East Africa [47, 48]. Despite these variations in prevalence of obesity and weight gain, the metabolic complications of dolutegravir may contribute to hepatic fat accumulation, resulting in the development of SLD among lean individuals [49, 50].

Furthermore, while there is paucity of SLD prevalence based on the CT criterion in sub-Saharan Africa, other studies have reported variation in prevalence depending on which criterion is used. For instance, the Multi-Ethnic Study of Atherosclerosis reported a prevalence of 16.9% based on liver to spleen attenuation ratio <1 [51]. In contrast, a separate study reported a prevalence of 40.9% by defining SLD as liver to spleen attenuation ratio <1 and/or a liver-spleen attenuation difference <1 HU [52]. Finally, a study using only an absolute liver attenuation <40 HU reported a prevalence of 10.0% [53].

Our study findings should be interpreted with limitations in mind. First, we evaluated SLD using CT images that, while part of the imaging protocol, may not have captured the entirety of the liver to fully assess for hepatic steatosis. While the use of CT imaging in detecting hepatic steatosis, by measuring liver CT attenuation and comparing it with spleen CT attenuation, has been validated as an accurate modality [29, 54], it may be limited in evaluating mild steatosis [55]. As such, our results may underestimate the true prevalence of SLD in this population. Second, information on alcohol misuse with AUDIT-C was by self-report from the participants, and it has been shown that there is underreporting on alcohol consumption particularly among PWH [56]. Third, as with all observational studies, we cannot eliminate the risk of confounding between HIV as our primary exposure and SLD as our primary outcome. We attempted to mitigate this with a study design that enrolled ambulatory PWH in long-term care in the public sector and population-based PWoH directly from the same communities as the PWH, and we attempted to measure other potential confounders of liver disease, including alcohol misuse, anthropometrics, and viral hepatitis infection. These findings were strengthened by use of a large representative cohort of PWH and the community-based PWoH as controls.

In conclusion, using noncontrast CT images, we found a low overall prevalence of SLD in Uganda but that HIV and use of ART were associated with an approximate doubling of the prevalence of SLD, even after removing individuals reporting alcohol misuse. In view of the limitations of CT in evaluation of SLD, these data should be corroborated with other diagnostic tools, such as transient elastography. Should our findings be confirmed, we recommend that screening for SLD be incorporated into HIV care programs for those with increased risk of SLD in this population.

## Supplementary Material

ofag325_Supplementary_Data

## Supplementary Data

Supplementary materials are available at *Open Forum Infectious Diseases* online. Consisting of data provided by the authors to benefit the reader, the posted materials are not copyedited and are the sole responsibility of the authors, so questions or comments should be addressed to the corresponding author.
