# Supplementary material for: Prevalence and Correlates of Steatotic Liver Disease Among People With HIV in Uganda
Source: Open Forum Infect Dis. 2026 May 27;13(6):ofag325. doi: 10.1093/ofid/ofag325 (PMC13262536; doi:10.1093/ofid/ofag325)
Supplement: ofag325_Supplementary_Data [file ofag325_supplementary_data.docx]

**Supplemental Table 1**: Correlates of SLD in those with negative AUDIT-C scores

| Factor | Unadjusted Model  OR (95% CI) | *P*-value | Adjusted Model*  OR (95% CI) | *P*-value |
| --- | --- | --- | --- | --- |
| HIV-serostatus |  |  |  |  |
| PWoH | REF | REF |  |  |
| PWH | 2.45 (0.85, 7.06) | 0.097 | 2.96 (1.00, 8.70) | 0.048 |
| Age, each ten years | 0.88 (0.42, 1.85) | 0.738 |  |  |
| Female | 0.96 (0.36, 2.52) | 0.927 |  |  |
| Non-HDL Cholesterol, each 10mg/dL | 1.15 (1.03, 1.28) | 0.011 | 1.13 (1.01, 1.27) | 0.033 |
| Waist circumference, each cm | 1.04 (1.00, 1.08) | 0.049 | 1.03 (0.99, 1.07) | 0.185 |
| Hemoglobin A1c, each % | 1.11 (0.81, 1.53) | 0.516 |  |  |
| Systolic blood pressure, each 10 mmHg | 1.07 (0.84, 1.36) | 0.590 |  |  |
| Diastolic blood pressure, each 10 mmHg | 1.18 (0.79, 1.75) | 0.417 |  |  |
| Body Mass Index (kg/m^2^) |  |  |  |  |
| 18.5 - <25 | REF | REF |  |  |
| <18.5 | 1.14 (0.13, 9.70) | 0.908 |  |  |
| 25 - <30 | 2.71 (0.92, 7.98) | 0.070 |  |  |
| >30 | 1.27 (0.25, 6.45) | 0.771 |  |  |

*Adjusted model: all variables who had a P-value of less than 0.25 in univariate/unadjusted model

**Supplemental Figure 1**: Crude prevalence of steatotic liver disease by FIB-4 Index, APRI score, and Hepatitis B Virus Infection;

**A** - FIB-4 Index; **B** - APRI score; **C** - Hepatitis B Virus Infection; Fibrosis-4 (FIB-4) index: FIB-4<1.45 - no fibrosis; FIB-4 >=1.45 - <=3.25 - indeterminate; FIB-4>3.25 - significant fibrosis; AST-to-platelet ratio index (APRI): APRI=<0.5 - no fibrosis; APRI>0.5 - <1.5 - indeterminate; APRI>=1.5 - significant fibrosis; HBV - Hepatitis B Virus; the bars display standard errors
